# Effects of Slaughter Age on the Quality of Gannan Yak Meat: Analysis of Edible Quality, Nutritional Value, and GC × GC‐ToF‐MS of the Longissimus Dorsi Muscle

**DOI:** 10.1002/fsn3.70381

**Published:** 2025-06-13

**Authors:** Xin Yu, Caiyun Li, Ziyi Zhao, Yubin Zhang

**Affiliations:** ^1^ College of Food Science and Engineering Gansu Agricultural University Lanzhou China

**Keywords:** edible quality, Gannan yak, GC × GC‐ToF‐MS analysis, nutritional value, slaughter age

## Abstract

Gannan yak meat is a natural green organic food with rich nutritional value. However, the slaughter age of Gannan yak in the market is older and has lower meat quality. In this study, yaks of different slaughtering ages were selected to assess the edible quality, nutritional value, and flavor characteristics of the longissimus dorsi (LD) muscle, and the optimal slaughtering age was identified based on the results. The results showed that the shear force (6.10 kgf), water loss rate (26.89%), cooking loss (23.71%), and drip loss (1.95%) of yak meat were significantly lower when the slaughter age was 2–4 years than in the other slaughter age groups (*p* < 0.05). Meanwhile, the cohesiveness (0.73%), elasticity (0.86), gumminess (61.71 mJ), monounsaturated fatty acid (0.4591 g/100 g), and polyunsaturated fatty acid contents (0.1232 g/100 g) of yak meat were significantly higher in the 2–4‐year slaughter age group (*p* < 0.05). Additionally, through GC × GC‐ToF‐MS analysis, a slaughter age of 2–4 years yielded yak meat with the highest content of key active flavor substances (e.g., 2‐nonenal, (E)‐, 2,3‐butanedione, etc.) and corresponding relative odor activity values (*p* < 0.05), making it more complex and conferring distinct flavor characteristics. In summary, meat obtained from 2 to 4‐year‐old Gannan yak was superior in terms of edible quality, nutritional value, and flavor characteristics and was more suitable for processing into high‐quality meat products.

## Graphical abstract

Gannan yak meat is a natural green organic food with rich nutritional value. However, the slaughter age of Gannan yaks in the market is older and has lower meat quality. The meat quality of Gannan yak aged 2–4 years is the best, and the content of monounsaturated fatty acids and polyunsaturated fats is the highest. The 2–4‐year‐old Gannan yak meat has more complex and distinct flavor characteristics.

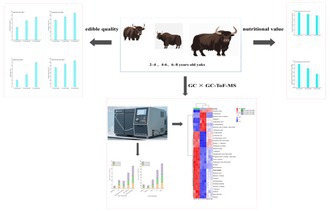

## 1 Introduction

As home to around 16 million yaks, China accounts for more than 95% of the global yak population. Although yak meat is a rare commodity in the international market, including Europe, it is highly valued by consumers for its flavor and rarity (Bai et al. 2023). Yaks thrive in the harsh alpine environments above 3000 m in altitude, having acclimated to the region's low oxygen and cold temperatures (Liu et al. 2023). This remarkable adaptability enables them to utilize the plateau's plentiful grassland resources for animal production, while also endowing their meat with high protein, low fat, a mellow flavor, and rich nutritional content (Li et al. 2024). In particular, meat from Gannan yaks—which are raised on natural pastures—is rich in protein, amino acids, and fatty acids, providing significant benefits in terms of disease resistance, cell vitality, and overall health. Hence, Gannan yak meat is considered a natural, green food (Ma et al. 2019). Furthermore, Gannan yaks are also recognized as a high‐quality local genetic resource in China (Niu et al. 2024).

With global economic development, the meat industry has grown rapidly, leading to increased consumer focus on meat quality (Kopuzlu et al. 2018). However, due to traditional customs, herders in the Gannan region are generally reluctant to sell their yaks (Degen et al. 2020). The herders often raise yaks to older ages rather than slaughtering them for meat at the optimal time, which can affect meat quality. Moreover, older slaughter ages can also increase feeding costs, threatening grassland sustainability (Bai et al. 2023). Accordingly, older slaughter ages may hinder the development of the yak industry. Therefore, standardized and normalized slaughter age guidelines are urgently required for Gannan yaks.

Studies have shown that slaughter age significantly affects the physical and chemical properties, nutritional content, and flavor compound composition of meat, although this effect varies with species (ruminants, such as cattle and sheep), breed, and the growth environment (Bai et al. 2023; Bureš and Bartoň 2012; Choi et al. 2023; Chen et al. 2022). For instance, Cho et al. (2015) found that the myoglobin content of Korean Hanwoo beef (longissimus dorsi muscle) increases with age, resulting in a deeper meat color. Meanwhile, Bai et al. (2023) observed that the tenderness and water‐holding capacity (WHC) of Datong yak meat (longissimus thoracis muscle) decrease significantly with age, possibly due to the unique muscle structure of yaks and their high‐altitude environment. It may also be because the crosslinking degree of connective tissue protein and myofibrillar protein in muscle increased significantly with age (Bakhsh et al. 2019). This structural change will lead to an increase in the shear force and hardness of aged yak meat, and a decrease in WHC, cohesiveness, gumminess, and elasticity (Gou et al. 2024). Furthermore, Choi et al. (2023) studied the effects of slaughter age (3–36 months) on meat from Black Korean Goats (LD) and found that its amino acid content increases with age, though no significant difference was observed across the ages of 12, 24, and 36 months. Other studies have also shown that in Holstein Frisian bulls, the content of saturated fatty acids (SFA) increases with age, while that of polyunsaturated fatty acids (PUFA) decreases (Diler et al. 2022). In contrast, other studies have found no significant impact of slaughter age on factors such as the cooking loss, water loss rate, shear force, and amino acid content of meat (Turan et al. 2021; Lorenzo et al. 2019).

In terms of flavor compounds, Chen et al. (2022) compared the quality of Xiangxi yellow cattle meat (LD) at different slaughter ages (6, 18, and 30 months) using gas chromatography–mass spectrometry (GC–MS). They found that the concentration of volatile flavor compounds in the meat of Xiangxi yellow cattle was higher when the slaughter age was 18 and 30 months. Similarly, Wang et al. (2021) used gas chromatography–ion mobility spectrometry (GC‐IMS) to study the effects of slaughter age (2, 6, and 12 months) on volatile flavor compounds in Jingyuan mutton (LD). Their findings showed that meat from 6‐month‐old lambs had the highest concentration of volatile compounds, while the levels of compounds such as 2‐nonenal, (E)‐, 2‐octenal, (E)‐, 1‐heptanol, 1‐octen‐3‐one, 1‐hexanol, and 1‐octen‐3‐ol decreased with increasing slaughter age. These findings suggest that the impact of slaughter age on meat flavor compounds also varies across species, possibly due to differences in fat content, enzyme activity, and metabolic pathways in muscle tissue. Notably, comprehensive two‐dimensional gas chromatography–time of flight mass spectrometry (GC × GC‐ToF‐MS) has been shown to offer higher sensitivity and resolution in flavor compound analysis. Studies by Rocha et al. (2013) and Shen et al. (2023) have demonstrated that GC × GC‐ToF‐MS can facilitate the identification of many more compounds than conventional GC, providing robust technical support for the in‐depth analysis of meat flavor.

While previous reports show that slaughter age can significantly affect meat quality (Bai et al. 2023; Choi et al. 2023), few studies have comprehensively analyzed the impact of slaughter age on the meat quality of Gannan yaks. Notably, there is no research on changes in the flavor quality of Gannan yak meat based on GC × GC‐ToF‐MS analysis. Therefore, this study systematically analyzed the edible quality, nutritional value, and flavor characteristics of yak meat at different slaughter ages to provide a theoretical foundation and technical support for the standardization of Gannan yak slaughter.

## 2 Materials and Methods

### 2.1 Material

All the animal procedures were carried out in accordance with the guidelines of the Chinese Animal Protection Commission and the Ministry of Agriculture of the People's Republic of China. The yak treatment procedure was approved by the Ethics Committee of Gansu Agricultural University (License No: GSAU‐Eth‐FSE‐2024‐006).

### 2.2 Animals

Based on field trips: Gannan area is located in the northeast of the Qinghai‐Tibet Plateau, with an altitude of about 3000 m. It has a typical alpine climate. The annual average temperature is low, and the temperature difference between day and night is large. The annual precipitation is 400–800 mm, mainly concentrated in July–September. The region is dominated by mountainous and meadow terrain, with rich natural grassland resources, providing a broad grazing space for yaks. Yaks here mainly use traditional grazing methods. This study was carried out in Gannan Prefecture from June 2023 to December 2024. During the period, the climatic conditions were stable, no extreme weather occurred, the ecological environment was good, the grassland ecosystem was complete, the water source was rich, and the air was fresh, which provided favorable conditions for the healthy growth of yaks.

Thirty healthy male yaks aged 2–4 years (A), 4–6 years (B), and 6–8 years (C) were selected from an industrial slaughter facility (Changxiang Meat Industry Company, Maqu, Gannan, China). The yaks were slaughtered according to Chinese livestock and poultry slaughter standards. Before slaughter, the yaks were fully rested and fasted (anhydrous) for 12 h. In addition, all yaks were raised in the same place with the same feed. After slaughter, the longissimus dorsi (LD) muscle was rapidly isolated from the yak carcasses, and excess fat and connective tissue were removed. The LD muscle was cut into small pieces, each weighing about 100 ± 20 g. The color, pH value, shear force, water loss rate, cooking loss, and drip loss of all LD samples were measured within 24 h post‐slaughter. The samples remaining were quickly frozen, then transported to the laboratory and stored at −80°C for subsequent analysis.

### 2.3 Color

The surface color of meat samples was measured using the CIE L*, a*, b* Clelab system (CR‐410, Konica Minolta). Within 24 h after slaughter (4°C), the L*, a*, and b* values were recorded in three different regions (upper, middle, and lower) of each meat sample (LD). The instrument was calibrated using a standard white ceramic plate before use.

### 2.4 pH

The pH value of meat samples was measured with a portable pH meter (DKK‐2100, TOADKK, Japan) as described by Bai et al. (2023), with slight adjustments. The pH_0_ of the sample was measured immediately after slaughter. The pH_0_ meter probe was inserted into the meat samples (LD) at three different randomized positions (upper, middle, and lower), and the pH value was recorded after the reading became stable.

### 2.5 Water Holding Capacity

#### 2.5.1 Cooking Loss

The method described by Li et al. (2011) was adopted, with slight modifications. First, the meat samples (LD) were weighed, and their weight was recorded as *M*_0_. Subsequently, the samples were placed in polyethylene bags and heated in an 80°C water bath. When the internal temperature of the meat samples hit 75°C, they were removed and allowed to cool down to ambient temperature. Then, the weight of these meat samples was recorded as M_1_. The cooking loss was calculated using the following formula:

(1) $$\text{Cooking Loss}=\left\{\left(M_{0}-M_{1}\right)/M_{0}\right\}\times 100\%.$$

#### 2.5.2 Drip Loss

The drip loss was measured using Xin et al.'s (2022) method. Weighed (*W*_0_) samples (LD) were suspended in inflatable polyethylene bags at 4°C for 24 h. Precautions were taken to ensure that the samples did not touch the sides of the bags. Then, the samples were removed, dried, and weighed again (*W*_1_). The drip loss was then calculated using the following formula:

(2) $$\text{Drip Loss}=\left\{\left(W_{0}-W_{1}\right)/W_{0}\right\}\times 100\%.$$

#### 2.5.3 Water Loss Rate

The method described by Bakhsh et al. (2019) was adopted, with minor modifications. About 5 g of each LD sample was accurately weighed (*M*_2_). The meat samples were covered with double‐layer gauze and 18 layers of filter paper (Munktell M68, Safelab, Beijing, China). Using a YYW‐2 strain‐controlled unlimited compression device, each meat sample was subjected to a pressure of 35 kg for 5 min. Finally, the pressure was removed, and the samples were weighed immediately (*M*_3_). The water loss rate was calculated with the following formula:

(3) $$\text{Water Loss}=\left\{\left(M_{2}-M_{3}\right)/M_{2}\right\}\times 100\%.$$

### 2.6 Shear Force

The method described by Li et al. (2011) was adopted, with slight modifications. After measuring the cooking loss of the sample, they were cut along the orientation of muscle fibers using a cylindrical sampler (Φ1.27 cm). Each meat column was placed vertically on the V‐shaped knife holder of a tenderness meter (C‐LM4, Harbin, China) and compressed at a speed of 10 mm/min. Each sample was tested at least thrice to ensure the reliability and repeatability of the data.

### 2.7 Texture Profile Analysis (TPA)

The method described by Kopuzlu et al. (2018) was adopted, with minor modifications. Each sample (LD) was placed in a cooking bag, which was subsequently placed inside a cooking pot. The sample was heated in an induction cooker at 1500 W. Meanwhile, the temperature at the center of each meat sample was measured using a puncture thermometer. When the central temperature reached 75°C, the meat was removed and cooled to room temperature. Tendons and fat were removed, and the samples were trimmed into smaller pieces sized 3.5 × 3.0 × 2.0 cm parallel to the direction of the muscle fibers using a stainless steel knife.

The cooked samples were placed on a texture analyzer (Lloyd Model AMETEK LS5, Lloyd Instruments Ltd., Largo, Florida, USA), ensuring that the muscle fibers were perpendicular to the direction of the probe. The samples were compressed twice and analyzed in the TPA mode. The specific conditions were as follows: the range of the force‐sensing element was 2000 N; the tPA probe model was FTC PT 2A‐50 mm; the initial distance between the probe and the surface of the sample was 30 mm; the pre‐test rate was 30 mm/min; the detection rate was 30 mm/min; the post‐test rate was 30 mm/min; the minimum starting force was 0.5 N; the deformation percentage was 40%; and the data collection frequency was 100 Hz. TPA parameters were obtained using force–time curves. In this experiment, five parameters—hardness, cohesiveness, elasticity, gumminess, and chewiness—were evaluated.

### 2.8 Sensory Analysis

As described by Kopuzlu et al. (2018), we trained a sensory evaluation team of 10 individuals (5 men and 5 women) in a standardized manner. Each participant provided informed consent, and the sensory evaluation protocol was approved by the Ethics Committee of Gansu Agricultural University. The samples were randomly placed on disposable pieces of cardboard. Yak meat was assessed based on four attributes: tenderness, juiciness, aroma, and taste. These characteristics were measured using a 9‐point descriptive scale: (9 = very tender, 1 = very hard; 9 = very juicy, 1 = very dry; 9 = extremely strong beef aroma, 1 = extremely weak aroma; and 9 = full‐bodied taste, 1 = bland taste).

Before sensory evaluation, meat samples (LD) were placed in plastic bags and cooked in a 90°C water bath until an internal temperature of 70°C was reached. After cooking, the samples were placed on a paper towel for 5 min to remove excess moisture. Subsequently, the meat was cut into smaller pieces, each weighing approximately 10 g, and then sent to the sensory panel for evaluation. Meat samples from different slaughter age groups were randomly assigned to panel members, who were tasked with evaluating the tenderness, juiciness, aroma, and taste of yak meat from these different groups. Each sample was tested three times. To cleanse their palates, panelists rinsed their mouths with plain water between each round of evaluation.

### 2.9 Conventional Components

The contents of moisture, ash, fat, and protein in the samples were determined using the Chinese standards GB/T 5009.3‐2016, GB/T 5009.4‐2016, GB/T 5009.6‐2016, and GB/T 5009.5‐2016, respectively.

### 2.10 Free Amino Acids (FAA)

Using the methodology proposed by Chen et al. (2022), the FAA in the meat samples was examined. A 0.5 g piece of meat (LD) (100°C, 5 min) was homogenized in 5 mL of ultrapure water using an ULTRA TURRAX disperser for 1 min at 10,000 × *g*. The sample was then centrifuged at 12,000 × *g* and 4°C for 15 min, and the supernatant was collected. An additional 2 mL of ultrapure water was added, and the extraction process was repeated. The supernatants were combined, and 1 mL of a 30% (m/v) zinc acetate solution was added. The mixture was then diluted to 10 mL with water. After another round of centrifugation at 12,000 × *g* for 15 min, the top liquid layer was filtered through a 0.22‐μm membrane. This was followed by derivatization.

For pre‐column derivatization, 0.4 M borate buffer (pH 10.2) was used with o‐phthalaldehyde as the primary derivatizing agent for amino acids, and 9‐fluorenylmethyl chloroformate served as the secondary derivatizing agent. Sample analysis was conducted using an Agilent 1100 Series High‐Performance Liquid Chromatography (HPLC) system. Primary amino acids were quantified using valine as the internal standard, while secondary amino acids were quantified using sarcosine as the internal standard. Glutamic acid, asparagine, and tryptophan were used as external standards (Agilent Technology Inc., Waldbronn, Germany).

### 2.11 Fatty Acids

Based on the method described by Chen et al. (2022), with slight modifications, 5 g of minced meat (LD) was freeze‐dried and powdered. Next, 0.5 g of meat powder was weighed and mixed with 1.0 mL of 1.0 mg/mL tridecanoic acid glyceride. Methanol was used as the standard, and 4 mL of a solvent composed of benzene and petroleum ether (1:1) was used to extract the total lipids from meat samples. After extraction, the lipid extract was combined with 4 mL of methanol solution containing 0.4 M KOH and vortexed to obtain fatty acid methyl esters. Once the methylation reaction was complete, 10 mL of saturated NaCl was added to the samples, followed by another round of vortexing to facilitate solution stratification. The upper liquid containing the fatty acid methyl esters was then collected and mixed with 1.0 g of Na_2_SO_4_ before centrifugation at 800 × *g* for 5 min. After centrifugation, 0.1 mL of the fatty acid methyl ester extract was diluted with n‐hexane to a volume of 1 mL and transferred to a 2 mL glass bottle. This sample was then analyzed using GC–MS.

GC–MS analysis was conducted using a Shimadzu GC–MSQP2010 system (Shimadzu, Tokyo, Japan) with a DB‐5MS column (30 m length, 0.25 mm internal diameter, 0.25 μm film thickness; Agilent Technologies Co., Palo Alto, CA, USA). A 2 μL aliquot of the prepared fatty acid methyl ester extract was injected at a temperature of 250°C. The injector was set to the split mode with a split ratio of 10:1. Helium (99.9999%) was used as the mobile phase, with a flow rate of 1 mL/min. The column oven temperature was maintained at 50°C for 1 min, then increased to 160°C at 20°C/min, held at 160°C for 1 min, elevated to 250°C at 10°C/min, and maintained at 250°C for 10 min. After heating to 250°C, the scan was conducted in the electron impact mode (70 eV) at a scan range of m/z 33–550. A mixed standard containing 37 fatty acid methyl esters was used as the reference for detection and measurement (Supelco Inc., Bellefonte, PA, USA).

### 2.12 GC × GC‐ToF‐MS Analysis

#### 2.12.1 Extraction of Flavor Substances

Configuration of internal standard solution: First, 100 mg of deuterated n‐hexanol‐d13 (purity 98.5%; c/D/N ISOTopes, Canada) was dissolved in 50% ethanol to prepare an internal standard solution with a concentration of 10 mg/L. This solution was then stored at 4°C for cryopreservation.

A 1 g sample of meat (LD) was placed into a 10 mL headspace vial, followed by 10 μL of the internal standard solution. The sample was then incubated at 60°C for 40 min. Before sample uptake, a solid‐phase microextraction (SPME) extraction head was conditioned at 250°C for 5 min and then transferred to the heating chamber. The sample was adsorbed at 60°C for 30 min. After the adsorption process was completed, the extraction head was moved to the gas chromatography inlet for desorption at 250°C for 5 min. Once the injection was finished, the extraction head was conditioned again at 270°C for 10 min. Finally, 10 μL of n‐alkanes (1000 mg/L; Sigma Aldrich Trading Co. Ltd.) was placed into a 20 mL headspace sampling bottle for incubation, extraction, and final injection (Grabež et al. 2019).

#### 2.12.2 Chromatographic Mass Spectrometry Analysis Conditions

The GC × GC‐ToF‐MS system includes an Agilent 8890A chromatograph, a two‐stage jet modulator, a split/splitless injection module, and a high‐resolution ToF mass spectrometer (Agilent Technologies, Palo Alto, CA, USA). For GC × GC‐ToF‐MS, high‐purity helium (99.9999%) was used as the carrier gas at a fixed flow rate of 1.0 mL/min. The one‐dimensional chromatographic column used was DB‐Heavy Wax (30 m × 250 μm × 0.5 μm; Agilent, USA). The temperature was initially held at 40°C for 3 min and subsequently increased to 250°C at a rate of 5°C per min for 5 min. Meanwhile, the two‐dimensional chromatographic column used was Rxi‐5Sil MS (2 m × 150 μm × 0.15 μm; Restek, USA). The temperature setting was 5°C higher than that of the one‐dimensional chromatographic column, and the modulator temperature was 15°C higher. The modulation period was 4.0 s, and the inlet temperature was 250°C (Li et al. 2020).

### 2.13 Statistical Analysis

All experiments were repeated at least three times. Microsoft Excel 2010 was used for data processing, and the results were expressed as the mean ± standard deviation (*n* = 30). One‐way analysis of variance was performed using SPSS Statistics 25 software, with the post hoc Duncan test applied for significance analysis. *p* < 0.001 indicated that the difference was extremely significant, while *p* < 0.05 indicated that the difference was significant. Finally, Origin 2022 software was used for preparing plots and mapping.

To further analyze the differences in volatile flavor substances among different groups, Chroma ToF (V4.3x, LECO, San Jose, USA) software was applied for data processing. To compare data across different orders of magnitude, the data were normalized based on internal standards (Dunn et al. 2011). Subsequently, the partial least squares discriminant analysis (PLS‐DA) model and *t*‐test (*p* < 0.05) were used to identify volatile flavor substances with variable importance in projection (VIP) thresholds greater than 1. These substances were deemed to show significant intergroup differences (Boulesteix and Strimmer 2007).

In addition, as described by Luo et al. (2022), with slight alterations, the relative odor activity values (ROAVs) were used to accurately identify and determine the main volatile flavor components that show significant intergroup differences and affect meat flavor. First, the component with the largest contribution to the overall flavor of the sample was defined, and its ROAV was set to 100. Then, the ROAV values of other volatile components were calculated using the following formula:

(4) $$\text{ROAV}=100\times \left(\text{PeakB}/\mathrm{TB}\right)/\left(\text{PeakA}/\mathrm{TA}\right),$$

where PeakB and TB represent the normalized quantitative value and sensory threshold of the test substance, while PeakA and TA represent the normalized quantitative value and sensory threshold of the substance with ROAV = 100, respectively.

## 3 Results and Discussion

### 3.1 Effects of Slaughter Age on the Edible Quality of Gannan Yak Meat

#### 3.1.1 Physicochemical Indexes

Compared to other sensory attributes, meat color more intuitively reflects the quality of chilled fresh meat and directly influences consumers' purchasing decisions (Ruedt et al. 2023). As shown in Table 1, as the slaughter age of yaks increased, the L* value exhibited a significant decrease, while both the a* and b* values showed a notable increase (*p* < 0.05). Previous studies have revealed that heme iron levels are negatively correlated with meat brightness (L* value), and the increase in slaughter age leads to reduced meat brightness. This change may be due to the increase in heme iron content with age (Bureš and Bartoň 2012). Meanwhile, Silva et al. (2019) showed that an increase in slaughter age leads to increased myoglobin content in muscle tissues, thus causing a notable rise in the a* value of yak meat. In addition, Zhang et al. (2022) discovered a significant positive correlation between intramuscular fat content and the b* value of meat. Therefore, the b* value of Gannan yak meat increased with age in this study due to the gradual increase in intramuscular fat deposition (Choi et al. 2023). Li et al. (2011) examined how slaughter age affects the quality characteristics of meat from Qinchuan cattle. They discovered that the a* value increased significantly and the L* value decreased significantly with the increase in slaughter age, consistent with our findings.

**TABLE 1:** Effects of slaughter age on the *longissimus dorsi* physicochemical indexes of Gannan yaks.

Age	Color traits			pH_0_	Shear force (kg f)	Rate of water loss (%)	Water binding capacity	
	L*	a*	b*				Cooking loss (%)	Drip loss (%)
2–4‐year‐old yaks	32.83 ± 0.12^a^	10.34 ± 0.03^c^	6.41 ± 0.17^c^	6.39 ± 0.07^a^	6.10 ± 0.16^c^	26.89 ± 0.47^c^	23.71 ± 0.17^c^	1.95 ± 0.18^c^
4–6‐year‐old yaks	28.03 ± 0.11^b^	12.40 ± 0.06^b^	7.54 ± 0.20^b^	6.40 ± 0.07^a^	9.23 ± 0.26^b^	31.96 ± 0.59^b^	27.69 ± 0.15^b^	3.44 ± 0.25^b^
6–8‐year‐old yaks	26.01 ± 0.14^c^	15.44 ± 0.04^a^	9.13 ± 0.24^a^	6.39 ± 0.06^a^	10.96 ± 0.17^a^	36.95 ± 0.33^a^	32.79 ± 0.12^a^	6.87 ± 0.29^a^
*p*	***	***	***	ns	***	***	***	***

*Note:* ^a,b,c^Means within a column with different superscripts differ significantly (*p* ≤ 0.05); ns = *p* > 0.05; ⁎⁎⁎*p* ≤ 0.001; values are presented as mean ± standard deviation, with *n* = 10 per group.

Shear force directly reflects the tenderness of meat; as the shear force value increases, the tenderness of meat decreases (Xin et al. 2022). Table 1 shows that the shear force value of yak meat increased significantly with the increase in slaughter age (*p* < 0.05). This could be attributed to the enlargement of muscle fiber diameter in yaks with age (Bakhsh et al. 2019). Furthermore, connective tissues like collagen and elastin, both of which become tighter with age, reduce meat tenderness. Bai et al. (2023) explored the effects of slaughter age on the properties of Datong yak meat, discovering a notable decline in muscle tenderness as the animals aged. Their results were in line with the findings of the present study. In addition, this study also found that there was no significant difference in the pH_0_ value of yak meat at all ages (*p* > 0.05), which was attributed to its unique physiological characteristics and high‐altitude ecological environment (Liu et al. 2023). Zi et al. (2004) found that there was no significant difference in the pH value of yak meat at different ages, and it was higher than that of pork and beef, which was consistent with the results of this study.

Water loss rate, cooking loss, and drip loss are important indicators for assessing the processing quality of meat (Cho et al. 2015), while the latter two parameters can provide insights into its WHC (Xin et al. 2022). As shown in Table 1, the water loss rate, cooking loss, and drip loss of yak meat increased significantly with slaughter age (*p* < 0.05). This may be because the gap between muscle tissues expands with aging, and the water loss in the muscle tissues increases, increasing the water loss rate and drip loss of yak meat (Bai et al. 2023). In addition, with the increase in slaughter age, protein denaturation in the muscle is enhanced, and the crosslinking effect of collagen also increases. Collectively, these changes decrease the WHC of the muscle tissue, thus increasing the water loss during cooking and significantly increasing the cooking loss (Schönfeldt and Strydom 2011). Similarly, Gou et al. (2024) studied the effect of slaughter age on the quality characteristics of Qinghai yak meat and found that the water loss rate, cooking loss, and drip loss increased significantly with age. In summary, 2–4‐year‐old Gannan yaks are more suitable for slaughter, and the meat from these animals is optimal for further processing.

#### 3.1.2 Texture Profile Analysis

Meat texture is an important determinant of consumer preference and satisfaction (Pu et al. 2023). Table 2 shows that with the increase in slaughter age, the hardness and chewiness of yak meat increased significantly, while the cohesiveness, elasticity, and gumminess decreased significantly (*p* < 0.05). This may be because the content of connective tissue (such as collagen) and the degree of cross‐linking in yak muscle increased significantly with age, while the moisture content decreased and myoglobin content rose (Bai et al. 2023). These changes collectively induced a significant increase in muscle hardness and chewiness and a significant reduction in cohesiveness, elasticity, and gumminess (Gou et al. 2024). Studies have demonstrated that the hardness, chewiness, and elasticity of meat can affect consumer acceptance. In particular, Chinese people show a preference for meat products with low hardness and chewiness as well as good elasticity (Pu et al. 2023). Therefore, Gannan yak meat obtained at a slaughter age of 2–4 years appears to be more acceptable to consumers.

**TABLE 2:** Effects of slaughter age on TPA of *longissimus dorsi* muscle in Gannan yaks.

Age	Hardness (N)	Cohesiveness (%)	Elasticity	Gumminess (mJ)	Chewiness (mJ)
2**–**4‐year‐old yaks	41.24 ± 0.84^c^	0.73 ± 0.02^a^	0.86 ± 0.03^a^	61.71 ± 1.99^a^	50.77 ± 0.97^c^
4**–**6‐year‐old yaks	72.23 ± 0.58^b^	0.65 ± 0.01^b^	0.71 ± 0.02^b^	47.69 ± 1.84^b^	58.61 ± 0.79^b^
6**–**8 year‐old yaks	93.66 ± 0.70^a^	0.58 ± 0.02^c^	0.53 ± 0.04^c^	26.89 ± 1.76^c^	69.81 ± 0.64^a^
*p*	***	***	***	***	***

*Note:* ^a,b,c^Means within a column with different superscripts differ significantly (*p* ≤ 0.05); ⁎⁎⁎*p* ≤ 0.001; values are presented as mean ± standard deviation, with *n* = 10 per group.

#### 3.1.3 Effect of Slaughter Age on Sensory Evaluation of Gannan Yak Meat

**FIGURE 1:**
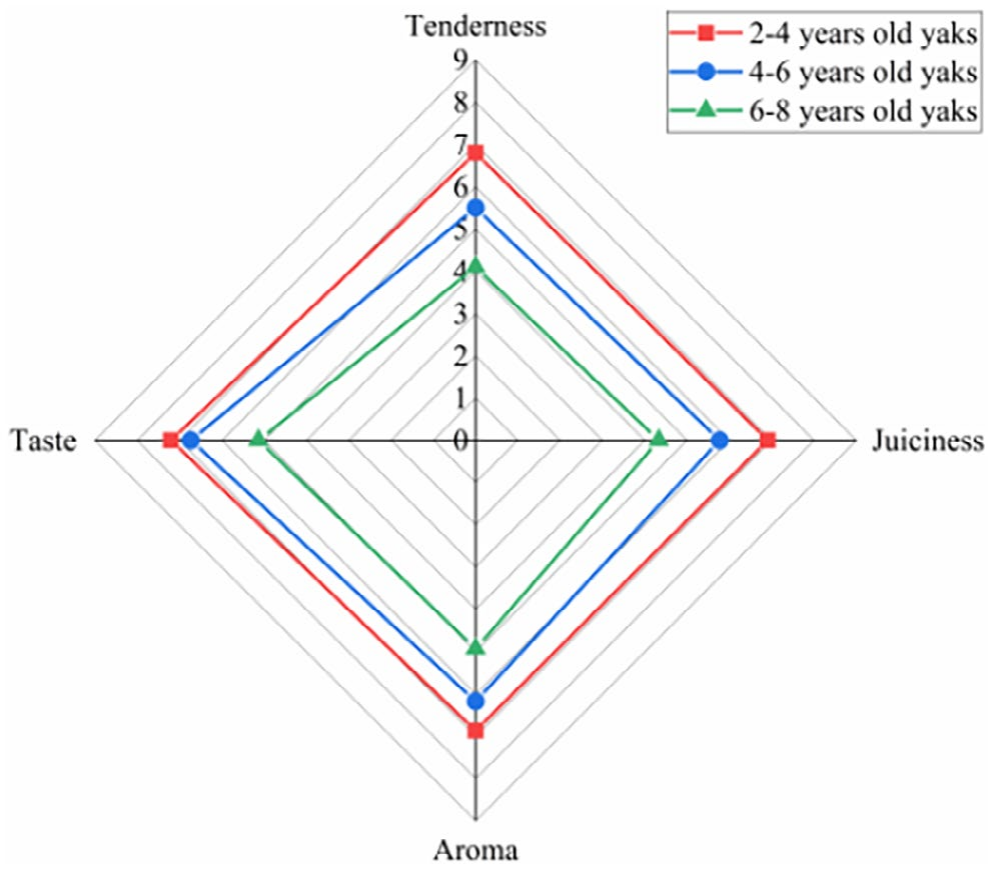
Radar map of sensory evaluation of longissimus dorsi muscle of Gannan yaks at different slaughter ages.

Sensory evaluation can intuitively reflect consumers' acceptance of meat. As shown in Figure 1, the tenderness, juiciness, flavor, and taste of meat were the highest at a slaughter age of 2–4 years and subsequently decreased with the increase in slaughter age. This may be due to the age‐related increase in the collagen content and degree of crosslinking in muscle tissue, resulting in a denser meat structure. Additionally, the integrity of cell membranes and myofibrillar proteins decreases with age, thereby reducing the tenderness and juiciness of meat (Bai et al. 2023). In addition, studies have shown that the content of malondialdehyde (MDA) in beef increases significantly with the increase in slaughter age, accelerating lipid oxidation and causing the deterioration of meat flavor and taste (Cho et al. 2015; Shahidi and Hossain 2022). Kopuzlu et al. (2018) studied the effect of slaughter age on the quality characteristics of eastern Anatolian beef and found that the tenderness, juiciness, flavor, and acceptability of the meat decreased significantly with the increase in slaughter age (19, 25, and 27 months), in line with the results of the present study. Therefore, Gannan yak meat obtained at a slaughter age of 2–4 years may be more popular with consumers.

### 3.2 Effects of Slaughter Age on the Nutritional Value of Gannan Yak Meat

#### 3.2.1 Basic Components

As observed in Table 3, an increase in slaughter age led to a significant reduction in the moisture content and a significant rise in the fat and protein content of yak meat (*p* < 0.05). This may be because fat and protein gradually accumulate in yak muscle with age (Bakhsh et al. 2019). In addition, with the increase in age, the gap between muscle tissues expands, resulting in a loose tissue structure and a decrease in water retention (Bai et al. 2023). Choi et al. (2023) found that as slaughter age increases, the water content of meat from Korean black goats decreases considerably, while the fat percentage shows a significant increase. Meanwhile, Li et al. (2011) explored the impact of slaughter age on the meat quality traits of Qinchuan cattle and found a significant decline in muscle moisture content with increasing slaughter age. Additionally, the levels of fat and protein in meat rose significantly, whereas the ash content remained relatively unchanged. These findings agree with the outcomes obtained in the present study.

**TABLE 3:** Effects of slaughter age on basic components of longissimus dorsi muscle of Gannan yaks meat.

Age	Moisture content (%)	Ash content (%)	Lipid content (%)	Protein content (%)
2–4‐year‐old yaks	73.13 ± 0.19^a^	1.059 ± 0.0017^a^	1.06 ± 0.02^c^	24.88 ± 0.11^c^
4–6‐year‐old yaks	67.84 ± 0.27^b^	1.062 ± 0.0050^a^	1.65 ± 0.08^b^	26.94 ± 0.14^b^
6–8‐year‐old yaks	63.59 ± 0.16^c^	1.066 ± 0.0023^a^	2.03 ± 0.05^a^	27.87 ± 0.18^a^
*p*	***	ns	***	***

*Note:* ^a,b,c^Means within a column with different superscripts differ significantly (*p* ≤ 0.05); ns = *p* > 0.05; ⁎⁎⁎*p* ≤ 0.001; values are presented as mean ± standard deviation, with *n* = 10 per group.

#### 3.2.2 Free Amino Acids

FAA are crucial bioactive constituents of meat. Additionally, the variety and concentrations of FAA also affect the sensory characteristics of meat products (Choi et al. 2023). As shown in Table 4, the total amino acid content of yak meat did not change significantly (*p* > 0.05) with the increase in slaughter age. Evidence shows that the concentrations of amino acids in meat generally remain stable and are largely unaffected by variables such as age, breed, and the type of cut (Zi et al. 2004). Glutamic acid and aspartic acid impart an umami taste; serine, alanine, and glycine are linked to sweetness; while arginine, valine, tyrosine, leucine, and phenylalanine provide bitterness (Xiao et al. 2020). In this study, the contents of glutamic acid, aspartic acid, alanine, and serine were found to decline significantly as the slaughter age increased, whereas the contents of arginine, valine, tyrosine, leucine, and phenylalanine increased (*p* < 0.05). This was probably due to changes in protein substitution and protein synthesis rates during animal growth, altering the individual content of specific FAA but not their overall levels (Watanabe et al. 2004). The rise in the levels of bitter‐tasting FAA is known to impair the sensory attributes of meat by neutralizing the intended flavor elements (Choi et al. 2023). Li et al. (2011) discovered that slaughter age does not significantly influence the amino acid content of Qinchuan beef. The predominant amino acid in Qinchuan beef was found to be glutamic acid, followed by aspartic acid, lysine, and leucine. Lysine emerged as the essential amino acid with the highest concentration, while glutamic acid was the most abundant non‐essential amino acid. The results from their study align with the findings of the present research. Thus, the amino acid composition and content of yak meat appear to be the best at a slaughter age of 2–4 years.

**TABLE 4:** Effect of slaughter age on FAA content in *longissimus dorsi* of Gannan yaks.

Amino acid content (g/100 g)	Age			*p*
	2–4‐year‐old yaks	4–6‐year‐old yaks	6–8‐year‐old yaks	
Aspartic acid (Asp)	2.02 ± 0.03^a^	1.91 ± 0.05^b^	1.83 ± 0.04^b^	**
Glutamic acid (Glu)	3.53 ± 0.03^a^	3.47 ± 0.03^a^	3.37 ± 0.05^b^	**
Serine (Ser)	0.90 ± 0.03^a^	0.83 ± 0.01^b^	0.80 ± 0.02^c^	***
Glycine (Gly)	0.94 ± 0.04^a^	0.93 ± 0.06^a^	0.90 ± 0.06^a^	ns
Histidine (His)	0.95 ± 0.03^a^	0.94 ± 0.03^a^	0.93 ± 0.04^a^	ns
Arginine (Arg)	1.18 ± 0.04^c^	1.45 ± 0.04^b^	1.62 ± 0.07^a^	***
Alanine (Ala)	1.25 ± 0.02^a^	1.20 ± 0.05^ab^	1.15 ± 0.05^b^	ns
Proline (Pro)	0.84 ± 0.04^a^	0.83 ± 0.05^a^	0.82 ± 0.04^a^	ns
Cystine (Cys)	0.35 ± 0.02^c^	0.44 ± 0.02^b^	0.49 ± 0.02^a^	***
Tyrosine (Tyr)	0.67 ± 0.04^c^	0.75 ± 0.02^b^	0.86 ± 0.03^a^	***
Threonine (Thr)^1^	1.02 ± 0.04^a^	1.01 ± 0.03^a^	1.00 ± 0.06^a^	ns
Tryptophan (Trp)^1^	0.21 ± 0.02^a^	0.19 ± 0.01^a^	0.14 ± 0.02^b^	**
Valine (Val)^1^	0.93 ± 0.03^b^	0.98 ± 0.03^b^	1.06 ± 0.03^a^	**
Methionine (Met)^1^	0.52 ± 0.05^a^	0.51 ± 0.11^a^	0.54 ± 0.02^a^	ns
Isoleucine (Ile)^1^	0.97 ± 0.06^a^	0.95 ± 0.05^a^	0.95 ± 0.06^a^	ns
Leucine (Leu)^1^	1.76 ± 0.11^a^	1.72 ± 0.08^a^	1.71 ± 0.08^a^	ns
Phenylalanine (Phe)^1^	0.96 ± 0.03^c^	1.02 ± 0.03^b^	1.07 ± 0.02^a^	**
Lysine (Lys)^1^	2.58 ± 0.02^a^	2.42 ± 0.06^b^	2.29 ± 0.04^c^	***
Total amino acids	21.58 ± 0.06^a^	21.56 ± 0.01^a^	21.54 ± 0.08^a^	ns
Essential amino acids	8.95 ± 0.13^a^	8.81 ± 0.09^a^	8.76 ± 0.13^a^	ns
Non‐essential amino acids	12.63 ± 0.11^a^	12.75 ± 0.08^a^	12.78 ± 0.05^a^	ns

*Note:* ^a,b,c^Means within a row with different superscripts differ significantly (*p* ≤ 0.05); ns = *p* > 0.05; ⁎⁎*p* ≤ 0.01; ⁎⁎⁎*p* ≤ 0.001; values are presented as mean ± standard deviation, with *n* = 10 per group.

^1^ Indicates essential amino acids.

#### 3.2.3 Fatty Acids

Fatty acids play an important role in maintaining human health (Choi et al. 2023), and meat is an important source of fatty acids. As shown in Table 5, with the increase in yak slaughter age, the content of SFA increased significantly, while that of unsaturated fatty acids (UFA) showed a significant decrease (*p* < 0.05). This could be attributed to the increase in the intramuscular fat content with age, which has an impact on the composition of fatty acids in meat. Notably, the ratio of neutral lipids to phospholipids affects the fatty acid composition of meat and manifests as an increase in SFA and a decrease in UFA (Lorenzo et al. 2019). Previous studies have shown that with the increase in slaughter age, the content of SFA in Holstein bull meat increases significantly, whereas the content of UFA decreases, consistent with the results of this study (Diler et al. 2022).

**TABLE 5:** Effects of slaughter age on fatty acid composition and content in *longissimus dorsi* muscle of Gannan yaks.

Fatty acid composition (g/100 g)	Age			*p*
	2–4‐year‐old yaks	4–6‐year‐old yaks	6–8‐year‐old yaks	
C8:0	0.0043 ± 0.0003^a^	0.0037 ± 0.0002^b^	0.0025 ± 0.0003^c^	***
C12:0	0.0014 ± 0.0002^c^	0.0032 ± 0.0003^b^	0.0059 ± 0.0004^a^	***
C14:0	0.0117 ± 0.0004^c^	0.0129 ± 0.0003^b^	0.0146 ± 0.0004^a^	***
C16:0	0.2221 ± 0.0003^c^	0.2235 ± 0.0002^b^	0.2245 ± 0.0003^a^	***
C18:0	0.1748 ± 0.0002^a^	0.1731 ± 0.0003^b^	0.1706 ± 0.0005^c^	***
C20:0	0.0092 ± 0.0004^c^	0.0107 ± 0.0004^b^	0.0118 ± 0.0003^a^	***
C22:0	0.0085 ± 0.0006^c^	0.0116 ± 0.0003^b^	0.0140 ± 0.0002^a^	***
C16:1^1^	0.0505 ± 0.0007^a^	0.0396 ± 0.0009^b^	0.0259 ± 0.0008^c^	***
C18:1^1^	0.3922 ± 0.0003^a^	0.3703 ± 0.0006^b^	0.3523 ± 0.0006^c^	***
C14:1n5^1^	0.0164 ± 0.0001^a^	0.0142 ± 0.0000^b^	0.00134 ± 0.0000^c^	***
C18:2^1^	0.0629 ± 0.0006^a^	0.0468 ± 0.0010^b^	0.0259 ± 0.0007^c^	***
C18:3^1^	0.0146 ± 0.0006^a^	0.0121 ± 0.0004^b^	0.0108 ± 0.0005^c^	***
C20:4^1^	0.0162 ± 0.0003^a^	0.0143 ± 0.0004^b^	0.0106 ± 0.0005^c^	***
C20:5^1^	0.0151 ± 0.0003^a^	0.0135 ± 0.0004^b^	0.0113 ± 0.0004^c^	***
C22:6^1^	0.0145 ± 0.0008^a^	0.0130 ± 0.0006^b^	0.0113 ± 0.0005^c^	**
Saturated fatty acids (SFA)	0.4321 ± 0.0003^c^	0.4388 ± 0.0004^b^	0.4439 ± 0.0003^a^	***
Unsaturated fatty acids (UFA)	0.5713 ± 0.0066^a^	0.5096 ± 0.0020^b^	0.4482 ± 0.0017^c^	***
Monounsaturated fatty acids (MUFA)	0.4591 ± 0.0010^a^	0.4242 ± 0.0013^b^	0.3916 ± 0.0014^c^	***
Polyunsaturated fatty acids (PUFA)	0.1232 ± 0.0020^a^	0.0996 ± 0.0008^b^	0.0700 ± 0.0003^c^	***
PUFA/SFA	0.2852 ± 0.0047^a^	0.2271 ± 0.0018^b^	0.1576 ± 0.0008^c^	***
MUFA/SFA	1.0624 ± 0.0021^a^	0.9667 ± 0.0038^b^	0.8822 ± 0.0035^c^	***
UFA/SFA	1.3222 ± 0.0153^a^	1.1614 ± 0.0055^b^	1.0097 ± 0.0042^c^	***

*Note:* ^a,b,c^Means within a row with different superscripts differ significantly (*p* ≤ 0.05); ns = *p* > 0.05; ⁎⁎*p* ≤ 0.01; ⁎⁎⁎*p* ≤ 0.001; values are presented as mean ± standard deviation, with *n* = 10 per group.

^1^ Denotes unsaturated fatty acids.

In addition, monounsaturated fatty acids (MUFA), the ratio of MUFA and PUFA to SFA (MUFA/SFA and PUFA/SFA), and the ratio of UFA to SFA (UFA/SFA) in yak meat all showed a significant downward trend with increasing slaughter age (*p* < 0.05). This was likely due to changes in the composition and activity of rumen microorganisms with age, especially the increase in microbial populations associated with biohydrogenation, which increased the biohydrogenation rate of UFA and thus enhanced the production of SFA (Lourenço et al. 2010). Similar findings were reported by Nogalski et al. (2018), who analyzed the fatty acid composition of Charolais and Holstein meat at different slaughter ages. They found that the PUFA/SFA and MUFA/SFA ratios decreased with the increase in slaughter age. The nutritional value of fatty acids in yak meat is typically determined based on the PUFA/SFA ratio (Wood et al. 2004). According to the WHO, a healthy diet must contain a PUFA/SFA ratio higher than 0.40. However, the ratio of PUFA to SFA in meat from ruminants does not meet these recommendations, mainly due to hydrogenation (Choi et al. 2023). As shown in Table 5, although the PUFA/SFA ratio in yak meat from 2 to 4‐year‐old yaks was less than 0.40, it was still significantly higher than that in meat from other slaughter age groups (*p* < 0.05). Chen et al. (2022) reported that the PUFA/SFA ratio of Angus cattle and Xiangxi cattle meat ranges from 0.19 to 0.33 across different slaughter ages, in line with the results of our study.

Notably, SFA increases the content of low‐density lipoprotein cholesterol (LDL) in the blood, which may lead to cardiovascular disease and impair health (Wood et al. 2004). Shramko et al. (2020) reported that palmitic acid (C16:0), a type of SFA, increases blood cholesterol levels, thereby having deleterious effects on health, while stearic acid (C18:0) has no significant effect on lipid metabolism. Meanwhile, studies have found that UFA can participate in many physiological processes in humans, including energy metabolism, lipid biosynthesis, the maintenance of membrane integrity, antioxidant responses, and apoptosis (Nogalski et al. 2018). In particular, oleic acid (C18:1) and linoleic acid (C18:2) can lower LDL levels in the blood and increase high‐density lipoprotein cholesterol (HDL) levels so as to maintain the balance of serum lipids, prevent cardiovascular diseases, and maintain human health (Shramko et al. 2020). In this study, with the increase in slaughter age, the content of palmitic acid (C16:0) and stearic acid (C18:0) in meat was found to increase significantly, while the content of oleic acid (C18:1) and linoleic acid (C18:2) showed a significant decrease (*p* < 0.05). These findings were in line with reports from Humada et al. (2012) and Kelava Ugarković et al. (2013), who studied the fatty acid content of meat from Tudanca, Simmental, Hereford, and Charolais cattle at different slaughter ages. In summary, from the perspective of fatty acid composition and nutritional value, the PUFA/SFA ratio (0.2880 ± 0.0048) in yak meat from 2‐ to‐year‐old yaks, and PUFA content (0.5713 ± 0.0066 g/100 g) was significantly higher than that of other age groups (*p* < 0.05). Therefore, the optimal age of slaughter for Gannan yaks is 2–4 years.

### 3.3 Effect of Slaughter Age on Volatile Flavor Compounds in Gannan Yak Meat

#### 3.3.1 Composition and Content of Volatile Flavor Compounds

**FIGURE 2:**
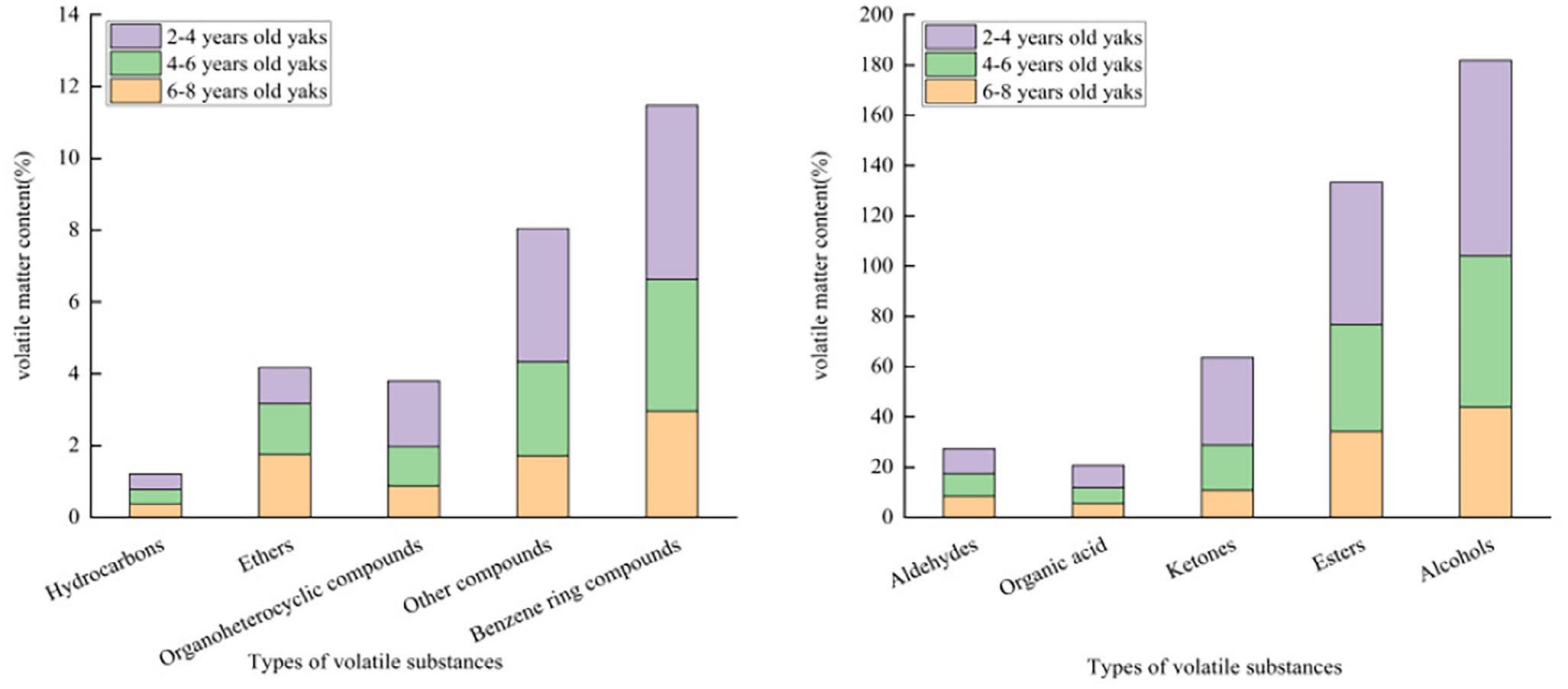
The accumulation of volatile flavor substances in the *longissimus dorsi* muscle of Gannan Yak at different slaughter ages.

GC × GC‐ToF‐MS combines one‐dimensional long‐column with two‐dimensional short‐column chromatography to provide more accurate data (Shen et al. 2023). As shown in Figure 2, the volatile fractions of Gannan yak meat were mainly composed of alcohols, esters, benzene ring compounds, ethers, aldehydes, hydrocarbons, ketones, organoheterocyclic compounds, organic acids, and other compounds. Notably, although the types of volatile compounds remained similar across different slaughter ages, there were significant variations in the contents of these compounds (*p* < 0.05). Table 6 shows that 88 volatile compounds were detected in Gannan yak meat obtained at slaughter ages of 2–4, 4–6, and 6–8 years, including 11 alcohols, 10 benzene ring compounds, two ethers, 11 aldehydes, four hydrocarbons, six ketones, 13 organoheterocyclic compounds, seven organic acids, 21 esters, and three other compounds. In addition, with the exception of hydrocarbons and ethers, the volatile compounds showed higher levels in meat obtained from the 2 to 4‐year group than in meat obtained from the other age groups (*p* < 0.05). Existing literature shows that the types of volatile compounds in livestock and poultry meat products remain similar across different slaughter ages but show notable variations in content (Wang et al. 2021). Chen et al. (2022) analyzed the volatile flavor compounds in meat from Xiangxi Yellow Cattle and Aberdeen Angus using GC–MS. The results showed that these two types of beef contained 44 volatile compounds, including alcohols, aldehydes, ketones, organic acids, esters, hydrocarbons, organoheterocyclic compounds, and other compounds, in line with the results of the present study.

**TABLE 6:** Effect of slaughter age on relative content of volatile flavor compounds in *longissimus dorsi* muscle of Gannan yak.

Number	Volatile compounds	Formula	1st Dimension time (s)	2nd Dimension time (s)	Content (%)			*p*
					2–4‐year‐old yaks	4–6‐year‐old yaks	6–8‐year‐old yaks	
Alcohols								
1	1‐Hexanol	C_6_H_14_O	1106.18	1.56	1.1157 ± 0.0034^a^	0.5773 ± 0.0533^b^	0.1271 ± 0.0261^c^	***
2	2‐Heptanol	C_7_H_16_O	1035.64	1.69	0.0360 ± 0.0022^b^	0.0358 ± 0.0034^b^	0.0435 ± 0.0020^a^	*
3	1‐Octanol	C_8_H_18_O	1504.90	1.68	0.8613 ± 0.0033^a^	0.6443 ± 0.0336^b^	0.5402 ± 0.0340^c^	***
4	1‐Heptanol	C_7_H_16_O	1309.92	1.62	0.8413 ± 0.0399^a^	0.2602 ± 0.0410^b^	0.0402 ± 0.0027^c^	***
5	Isopropyl Alcohol	C_3_H_8_O	330.81	1.38	0.7433 ± 0.0245^a^	0.4485 ± 0.0348^b^	0.3755 ± 0.0376^c^	***
6	1‐Octen‐3‐ol	C_8_H_16_O	1301.59	1.65	3.5381 ± 0.0443^a^	1.8373 ± 0.0280^b^	1.3983 ± 0.0224^c^	***
7	1‐Pentanol	C_5_H_12_O	899.94	1.50	0.1878 ± 0.0002^c^	0.2267 ± 0.0014^b^	0.3487 ± 0.0027^a^	***
8	1‐Propanol, 2‐methyl—	C_4_H_10_O	584.96	1.44	0.0843 ± 0.0037^a^	0.0473 ± 0.0038^b^	0.0360 ± 0.0014^c^	***
9	Ethanol	C_2_H_6_O	344.98	1.43	52.2925 ± 3.2155^a^	36.6700 ± 2.9470^b^	18.9955 ± 1.1534^c^	***
10	Linalool	C_10_H_18_O	1489.90	1.79	0.0766 ± 0.0113^a^	0.0178 ± 0.0029^c^	0.0506 ± 0.0059^b^	***
11	2‐Propanol, 2‐methyl—	C_4_H_10_O	302.20	1.42	17.9854 ± 0.3022^c^	19.5033 ± 0.0858^b^	21.8523 ± 0.2953^a^	***
Benzene ring compounds								
12	Benzeneacetic acid, ethyl ester	C_10_H_12_O_2_	1924.88	1.71	0.1816 ± 0.0217^c^	0.2482 ± 0.0240^b^	0.3723 ± 0.0233^a^	***
13	Phenol, 2‐methyl—	C_7_H_8_O	2259.86	1.30	0.0172 ± 0.0015^c^	0.0541 ± 0.0014^a^	0.0218 ± 0.0021^b^	***
14	Phenol, 3‐methyl—	C_7_H_8_O	2389.85	1.28	0.0143 ± 0.0004^a^	0.0020 ± 0.0004^c^	0.0084 ± 0.0007^b^	***
15	Biphenyl	C_12_H_10_	2249.86	1.76	0.0140 ± 0.0005^c^	0.0153 ± 0.0004^b^	0.0180 ± 0.0005^a^	***
16	Phenol, 4‐ethyl—	C_8_H_10_O	2509.84	1.30	0.0500 ± 0.0009^c^	0.0676 ± 0.0004^b^	0.1088 ± 0.0005^a^	***
17	Phenol	C_6_H_6_O	2264.86	1.25	0.3326 ± 0.0089^a^	0.2028 ± 0.0019^b^	0.1487 ± 0.0107^c^	***
18	Toluene	C_7_H_8_	504.97	1.89	1.1722 ± 0.0020^a^	0.9303 ± 0.0338^b^	0.4768 ± 0.0457^c^	***
19	p‐Cresol	C_7_H_8_O	2377.63	1.28	0.4847 ± 0.0753^b^	0.5133 ± 0.0265^b^	0.7185 ± 0.0691^a^	**
20	Terpinen‐4‐ol	C_10_H_18_O	1599.90	1.98	0.0169 ± 0.0006^a^	0.0168 ± 0.0002^a^	0.0159 ± 0.0003^b^	*
21	Benzene	C_6_H_6_	358.31	1.67	2.5618 ± 0.2587^a^	1.6121 ± 0.3238^b^	1.0731 ± 0.0143^c^	***
Ethers								
22	Ethane, 1,1‐diethoxy—	C_6_H_14_O_2_	296.65	1.97	0.0848 ± 0.0029^a^	0.0673 ± 0.0036^b^	0.0017 ± 0.0001c	***
23	Dimethyl sulfide	C_2_H_6_S	199.99	1.45	0.9263 ± 0.0399^c^	1.3427 ± 0.0572^b^	1.7531 ± 0.0323a	***
Aldehydes								
24	Benzeneacetaldehyde	C_8_H_8_O	1679.89	1.54	4.7985 ± 0.0367^a^	4.6460 ± 0.0466^b^	4.1643 ± 0.0218^c^	***
25	Hexanal	C_6_H_12_O	574.96	1.90	1.5996 ± 0.0334^a^	1.2830 ± 0.0275^b^	1.0235 ± 0.0032^c^	***
26	Methional	C_4_H_8_OS	1324.92	1.51	1.7354 ± 0.0169^a^	1.3243 ± 0.0068^b^	1.1942 ± 0.0071^c^	***
27	2,6‐Nonadienal, (E,Z)—	C_9_H_14_O	1574.90	1.86	0.0593 ± 0.0005^a^	0.0453 ± 0.0014^b^	0.0229 ± 0.0006^c^	***
28	2‐Octenal, (E)—	C_8_H_14_O	1274.92	1.94	0.1486 ± 0.0006^a^	0.1359 ± 0.0005^b^	0.1289 ± 0.0007^c^	***
29	Octanal	C_8_H_16_O	989.94	2.19	0.0792 ± 0.0037^a^	0.0166 ± 0.0015^b^	0.0069 ± 0.0004^c^	***
30	Butanal, 3‐methyl—	C_5_H_10_O	326.98	1.66	0.6675 ± 0.0166^a^	0.3822 ± 0.0062^b^	0.1846 ± 0.0184^c^	***
31	Heptanal	C_7_H_14_O	779.95	2.06	0.2766 ± 0.0163^c^	0.6277 ± 0.0335^b^	0.9694 ± 0.0203^a^	***
32	Propanal, 2‐methyl—	C_4_H_8_O	230.61	1.50	0.4382 ± 0.0244^b^	0.5688 ± 0.0221^a^	0.6180 ± 0.0490^a^	**
33	2‐Nonenal, (E)—	C_9_H_16_O	1479.91	2.02	0.0316 ± 0.0002^a^	0.0288 ± 0.0010^b^	0.0164 ± 0.0005^c^	***
34	2‐Hexenal, (E)—	C_6_H_10_O	849.95	1.76	0.0314 ± 0.0020^a^	0.0203 ± 0.0007^b^	0.0003 ± 0.0001^c^	***
Hydrocarbons								
35	Heptane	C_7_H_16_	176.66	2.02	0.0661 ± 0.0025^a^	0.0455 ± 0.0036^b^	0.0456 ± 0.0017^b^	***
36	Octane	C_8_H_18_	217.21	2.78	0.2766 ± 0.0434^a^	0.3028 ± 0.0151^a^	0.3134 ± 0.0266^a^	ns
37	Beta‐Myrcene	C_10_H_16_	724.95	2.88	0.0476 ± 0.0033^a^	0.0317 ± 0.0040^b^	0.0166 ± 0.0017^c^	***
38	Pentane, 2‐methyl—	C_6_H_14_	153.32	1.54	0.0432 ± 0.0013^a^	0.0203 ± 0.0013^b^	0.0022 ± 0.0005^c^	***
Ketones								
39	2‐Undecanone	C_11_H_22_O	1589.90	2.39	0.2479 ± 0.0025^a^	0.1995 ± 0.0058^b^	0.0938 ± 0.0032^c^	***
40	2,3‐Butanedione	C_4_H_6_O_2_	411.22	1.45	1.6543 ± 0.2269^c^	2.2864 ± 0.0807^b^	4.6042 ± 0.2374^a^	***
41	1‐Octen‐3‐one	C_8_H_14_O	1014.94	2.01	0.0750 ± 0.0038^a^	0.0582 ± 0.0032^b^	0.0490 ± 0.0065^b^	***
42	2‐Nonanone	C_9_H_18_O	1189.92	2.23	0.0443 ± 0.0032^b^	0.0618 ± 0.0039^b^	0.9448 ± 0.0420^a^	***
43	Acetone	C_3_H_6_O	234.36	1.40	32.6566 ± 6.4202^a^	15.3383 ± 3.448^b^	5.1548 ± 0.7445^c^	***
44	Cyclohexanone	C_6_H_10_O	1002.72	1.77	0.0728 ± 0.0010^a^	0.0485 ± 0.0020^b^	0.0244 ± 0.0038^c^	***
Organoheterocyclic compounds								
45	2(3H)‐Furanone, 5‐heptyldihydro—	C_11_H_20_O_2_	2639.83	1.70	0.0271 ± 0.0006^a^	0.0195 ± 0.0005^b^	0.0163 ± 0.0004^c^	***
46	Furan, 2‐pentyl—	C_9_H_14_O	869.94	2.41	0.4900 ± 0.0205^a^	0.2573 ± 0.0258^b^	0.1876 ± 0.0113^c^	***
47	Thiophene, 2‐pentyl—	C_9_H_14_S	1329.91	2.39	0.0629 ± 0.0021^a^	0.0444 ± 0.0012^b^	0.0255 ± 0.0028^c^	***
48	Butyrolactone	C_4_H_6_O_2_	1659.89	1.37	0.5541 ± 0.0180^a^	0.2312 ± 0.0126^b^	0.1661 ± 0.0215^c^	***
49	Pyrazine, trimethyl—	C_7_H_10_N_2_	1214.92	1.84	0.0168 ± 0.0003^c^	0.0240 ± 0.0003^b^	0.0348 ± 0.0004^a^	***
50	Furan, 2‐ethyl—	C_6_H_8_O	369.98	1.77	0.0823 ± 0.0053^a^	0.0577 ± 0.0040^b^	0.0385 ± 0.0018^c^	***
51	2H‐Pyran‐2‐one, tetrahydro‐6‐propyl—	C_8_H_14_O_2_	2219.30	1.57	0.0108 ± 0.0006^c^	0.0193 ± 0.0008^a^	0.0125 ± 0.0004^b^	***
52	Benzothiazole	C_7_H_5_NS	2194.86	1.55	0.1820 ± 0.0154^a^	0.1226 ± 0.0109^b^	0.0897 ± 0.0021^c^	***
53	Thiazole	C_3_H_3_NS	904.94	1.47	0.1497 ± 0.0042^a^	0.0891 ± 0.0030^b^	0.0498 ± 0.0020^c^	***
54	Pyrazine, methyl—	C_5_H_6_N_2_	939.94	1.61	0.0837 ± 0.0021^a^	0.0575 ± 0.0080^b^	0.0441 ± 0.0034^c^	***
55	Pyridine	C_5_H_5_N	759.951	1.57	0.0890 ± 0.0017^c^	0.1164 ± 0.0032^b^	0.1555 ± 0.0024^a^	***
56	Tetrahydrofuran	C_4_H_8_O	274.98	1.63	0.0593 ± 0.0020^a^	0.0510 ± 0.0014^b^	0.0493 ± 0.0021^b^	***
57	Thiophene, 3‐methyl—	C_5_H_6_S	594.96	1.80	0.0163 ± 0.0008^a^	0.0128 ± 0.0007^b^	0.0021 ± 0.0003^c^	***
Organic acids								
58	Butanoic acid	C_4_H_8_O_2_	1649.33	1.25	0.1874 ± 0.0023^a^	0.1090 ± 0.0019^b^	0.0596 ± 0.0014^c^	***
59	Nonanoic acid	C_9_H_18_O_2_	2504.28	1.37	7.9198 ± 0.3615^a^	4.6385 ± 0.4310^b^	3.1369 ± 0.4802^c^	***
60	Dodecanoic acid	C_12_H_24_O_2_	2994.81	1.61	0.0798 ± 0.0022^c^	0.1783 ± 0.0024^b^	0.2528 ± 0.0293^a^	***
61	n‐Decanoic acid	C_10_H_20_O_2_	2649.83	1.39	0.2462 ± 0.0297^c^	0.4262 ± 0.0255^b^	0.7939 ± 0.0354^a^	***
62	Hexanoic acid	C_6_H_12_O_2_	2019.87	1.29	0.1755 ± 0.0132^b^	0.5753 ± 0.0289^a^	0.6042 ± 0.0365^a^	***
63	Butanoic acid, 3‐methyl—	C_5_H_10_O_2_	1724.89	1.27	0.0167 ± 0.0003^b^	0.0085 ± 0.0007^c^	0.1958 ± 0.0023^a^	***
64	Heptanoic acid	C_7_H_14_O_2_	2189.86	1.31	0.3274 ± 0.0244^b^	0.3382 ± 0.0299^b^	0.4859 ± 0.0301^a^	***
Esters								
65	Hexadecanoic acid, ethyl ester	C_18_H_36_O_2_	2620.39	3.13	19.4011 ± 0.5016^a^	11.5244 ± 0.5837^b^	7.7196 ± 0.5304^c^	***
66	Decanoic acid, ethyl ester	C_12_H_24_O_2_	1662.11	2.79	9.4537 ± 0.1578^a^	7.2365 ± 0.1172^b^	4.1631 ± 0.1161^c^	***
67	Hexanoic acid, ethyl ester	C_8_H_16_O_2_	874.94	2.41	0.6010 ± 0.0353^a^	0.5072 ± 0.0366^b^	0.1593 ± 0.0177^c^	***
68	Nonanoic acid, ethyl ester	C_11_H_22_O_2_	1474.91	2.72	6.2142 ± 0.3227^b^	6.7906 ± 0.3522^b^	9.3389 ± 0.5060^a^	***
69	Butanedioic acid, diethyl ester	C_8_H_14_O_4_	1739.89	1.68	0.1469 ± 0.0071^c^	0.3782 ± 0.0156^a^	0.3221 ± 0.0241^b^	***
70	Propanoic acid, 2‐hydroxy‐, ethyl ester	C_5_H_10_O_3_	1105.76	1.46	6.8030 ± 0.2463^a^	5.7746 ± 0.4384^b^	4.0196 ± 0.3702^c^	***
71	Propanoic acid, 2‐methyl‐, ethyl ester	C_6_H_12_O_2_	389.98	1.98	0.1624 ± 0.0042^a^	0.0558 ± 0.0121^b^	0.0295 ± 0.0008^c^	***
72	Butanoic acid, 2‐methyl‐, ethyl ester	C_7_H_14_O_2_	519.97	2.25	0.0283 ± 0.0006^c^	0.0587 ± 0.0037^b^	0.0684 ± 0.0047^a^	***
73	Pentanoic acid, ethyl ester	C_7_H_14_O_2_	679.96	2.23	0.0190 ± 0.0021^c^	0.0631 ± 0.0037^a^	0.0363 ± 0.0016^b^	***
74	Benzenepropanoic acid, ethyl ester	C_11_H_14_O_2_	2089.87	1.79	0.0890 ± 0.0048^a^	0.0720 ± 0.0039^b^	0.0612 ± 0.0028^c^	***
75	Ethyl acetate	C_4_H_8_O_2_	296.09	1.56	7.5519 ± 0.3040^a^	4.5702 ± 0.3539^b^	2.7039 ± 0.2307^c^	***
76	2‐Propenoic acid, ethyl ester	C_5_H_8_O_2_	429.97	1.68	0.0467 ± 0.0040^c^	0.0695 ± 0.0035^b^	0.0977 ± 0.0047^a^	***
77	Butanoic acid, 3‐methyl‐, ethyl ester	C_7_H_14_O_2_	549.97	2.23	0.0310 ± 0.0025^c^	0.3712 ± 0.0266^b^	0.9110 ± 0.0329^a^	***
78	Heptanoic acid, ethyl ester	C_9_H_18_O_2_	1079.93	2.53	0.2533 ± 0.0046^c^	0.3359 ± 0.0034^b^	0.3645 ± 0.0053^a^	***
79	Butanoic acid, ethyl ester	C_6_H_12_O_2_	494.97	2.01	0.5356 ± 0.0192^a^	0.2996 ± 0.0138^b^	0.2538 ± 0.0104^c^	***
80	Propanoic acid, ethyl ester	C_5_H_10_O_2_	379.98	1.78	0.1224 ± 0.0023^c^	0.2614 ± 0.0025^b^	0.2733 ± 0.0038^a^	***
81	Tetradecanoic acid, ethyl ester	C_16_H_32_O_2_	2329.85	3.01	2.5488 ± 0.2703^a^	2.3575 ± 0.2143^a^	2.3254 ± 0.0987^a^	ns
82	Dodecanoic acid, ethyl ester	C_14_H_28_O_2_	2009.87	2.91	1.7608 ± 0.1052^a^	0.9127 ± 0.0780^b^	0.6229 ± 0.1137^c^	***
83	Diethyl phthalate	C_12_H_14_O_4_	2794.82	1.71	0.0118 ± 0.0003^c^	0.0156 ± 0.0013^b^	0.0194 ± 0.0006^a^	***
84	Ethyl formate	C_3_H_6_O_2_	244.98	1.43	0.8815 ± 0.0069^a^	0.8307 ± 0.0063^b^	0.7248 ± 0.0066^c^	***
85	Methyl formate	C_2_H_4_O_2_	209.99	1.34	0.0639 ± 0.0005^a^	0.0357 ± 0.0003^b^	0.0247 ± 0.0004^c^	***
Others								
86	Disulfide, dimethyl	C_2_H_6_S_2_	554.96	1.75	3.2816 ± 0.2598^a^	2.0603 ± 0.1297^b^	1.4038 ± 0.2881^c^	***
87	Dimethyl trisulfide	C_2_H_6_S_3_	1179.92	1.84	0.1949 ± 0.0024^a^	0.1121 ± 0.0046^b^	0.0515 ± 0.0087^c^	***
88	Trichloromethane	CHCl_3_	474.97	1.49	0.2336 ± 0.0306^b^	0.4409 ± 0.0512^a^	0.2685 ± 0.0451^b^	**

*Note:* ^a,b,c^Means within a row with different superscripts differ significantly (*p* ≤ 0.05); ns = *p* > 0.05; ⁎*p* ≤ 0.05; ⁎⁎*p* ≤ 0.01; ⁎⁎⁎*p* ≤ 0.001.

Esters are common volatile flavor compounds and are mainly produced by the reaction between alcohols and fatty acids during oxidation (Wang et al. 2021). These compounds typically have a lower odor threshold and can significantly enhance the fruity aroma of meat (García‐Béjar et al. 2020). Hexadecanoic acid, ethyl ester, and ethyl acetate are typical ester compounds that exude a strong fruity aroma (Watanabe et al. 2015). In this study, the contents of these compounds were found to be the highest in meat from 2 to 4‐year‐old yaks, decreasing thereafter with the increase in slaughter age. This may be because, with the increase of slaughter age, the content of MDA in beef will increase significantly, which will accelerate the oxidation of lipids, and the accumulation of lipid peroxidation products will lead to the oxidative consumption of ester precursors (such as free fatty acids), thereby reducing the production efficiency of ethyl palmitate and ethyl acetate (Cho et al. 2015; Shahidi and Hossain 2022). Wang et al. (2021) studied the volatile components in Jingyuan mutton and found that ethyl acetate showed the highest levels in young mutton, consistent with the results of our study.

Furan and its derivatives are important heterocyclic compounds. These compounds are mainly produced by the Maillard reaction and act as key contributors to the special aroma of meat (Kosowska et al. 2018). Among them, furan, 2‐ethyl‐, and furan, 2‐pentyl‐ are key flavor substances and are mainly derived from the degradation of thiamine. Notably, they enable the formation of the characteristic flavor of yak meat (Calkins and Hodgen 2007). In this study, the levels of these compounds were found to decrease with the increase in slaughter age. Thiazole compounds, which are also an important component of meat flavor, demonstrate low odor thresholds and a nutty aroma. Benzothiazole is the most representative thiazole compound (Halliwell and Chirico 1993), and its content in meat was found to be the highest in the 2–4‐year‐old yak group. The content of these three substances decreased with age, which may be due to the decrease of basal metabolic rate (BMR) and the shift of energy metabolism from oxidative phosphorylation to glycolysis with age (Brown et al. 2018). Studies have found that aging yaks are more dependent on gluconeogenesis and fat mobilization for energy supply, and thiamine, as a coenzyme of pyruvate dehydrogenase (PDH) and α‐ketoglutarate dehydrogenase (α‐KGDH), is consumed in large quantities in glucose metabolism (De Caterina et al. 2019).

Lactones, which are important flavor components, impart milky, fruity, nutty, and caramel flavors to food and interact with other volatile components to create a more harmonious overall flavor (Cameleyre et al. 2020; Cooke Née Brown et al. 2009). Butyrolactone, an important flavor‐producing lactone with caramel, sweet, and coconut flavor, has an aroma‐enhancing effect on meat (Nie et al. 2012). Its precursor is γ‐hydroxybutyrate (mainly converted from lactic acid), and the synthesis of γ‐hydroxybutyrate is mainly affected by the efficiency of glycolysis (Wang et al. 2021). In this study, its content was found to decrease significantly with the increase in yak slaughter age. This may be due to the down‐regulation of hypoxia‐inducible factor‐1α (HIF‐1α) in yaks due to oxidative stress or gene regulation with the increase of age, which further weakens the efficiency of glycolysis and reduces the accumulation of lactic acid, resulting in a decrease in butyrolactone content (He et al. 2016).

Aldehydes are mainly derived from the decomposition of alkoxy radicals during lipid oxidation or the Maillard reaction. The threshold of these compounds is generally low, and they easily synergize with other aroma components to endow meat with an oil and fruit flavor, thus having a decisive influence on the taste of meat (Liu et al. 2019). In this study, 11 aldehydes were detected in yak meat across different ages, among which benzeneacetaldehyde, methional, and hexanal showed the highest content. Benzeneacetaldehyde is generated via the Strecker degradation of amino acids (such as phenylalanine) or microbial metabolism and can impart almond flavor and honey aroma to meat products (Zhou et al. 2024). In this study, its content is likely due to changes in amino acid metabolic pathways (Gou et al. 2024). Hexanal, which is mainly produced by the oxidative degradation of C20:4 (arachidonic acid) and shows a grassy fragrance, showed the highest levels in the 2–4‐year yak meat group. This could be attributed to the decrease in C20:4 content with the increase in slaughter age (Zhou et al. 2024; García‐González et al. 2013). Methional and 2,6‐nonadienal, (E,Z)‐, which have a strong meat aroma and a strong fruity and fatty aroma (García‐González et al. 2013), respectively, showed the highest levels in meat obtained from yaks at 2–4 years of age. This may be related to the decrease in UFA in muscle tissues with the increase in slaughter age (Diler et al. 2022; Zhou et al. 2024). This is consistent with the previous research.

Alcohols are mainly derived from the oxidation of UFA and usually have plant, aromatic, and earthy flavors (Caporaso et al. 1977). Ethanol is typically the predominant alcohol in meat, endowing meat products with the flavors of herbs, wood, and fat (Lorenzo et al. 2013). 1‐Octen‐3‐ol is mainly generated via the oxidation of C18:2 (linoleic acid) and C20:4 (arachidonic acid). It has a mushroom and soil flavor, and its odor threshold is low; thus, it significantly contributes to the formation of meat flavor (Zhou et al. 2024). In this study, its content decreased with the increase in slaughter age, likely due to the age‐related decrease in the C18:2 and C20:4 content of yak meat. Meanwhile, hydrocarbons are usually produced by the decomposition of esters and show high flavor thresholds. Most alkanes have a weak aroma and are tasteless. Thus, although they have a certain modification effect, their contribution to the overall flavor of meat products is limited (Sun et al. 2020).

Ketones are derived from the oxidation of fats, the oxidation of alcohols, and the degradation of esters. Due to their high flavor threshold, ketones have little effect on the overall flavor of food products (O'Quinn et al. 2016). However, ketones are relatively stable and have a lasting aroma, which is typically floral (Wang et al. 2021). 1‐Octen‐3‐one, mainly formed by the oxidation of C18:2, showed the highest content in yak meat obtained at a slaughter age of 2–4 years, likely due to the decrease in C18:2 content with the increase in slaughter age (Zhou et al. 2024).

Organic acids are typically derived from metabolic processes such as lipid oxidation and degradation and other precursors. These metabolic pathways are controlled by key enzymes and proteins, such as cytochrome P450 2A6, kinesin‐like protein 12, and cytoplasmic sulfotransferase 1C1 (Mezgebo et al. 2017; Van Ba et al. 2013). The threshold of organic acids is relatively high, and their contribution to flavor is relatively limited. In this study, a total of seven organic acids were identified in yak meat across different ages. Among them, the content of nonanoic acid was highest at 2–4 years and decreased with the increase in slaughter age, showing sweet, grass, and fat flavor (Mezgebo et al. 2017). In contrast to organic acids, most ethers can produce a pleasant aroma, with nitrogen‐ and sulfur‐containing ethers having a lower aroma threshold, mainly due to the Maillard reaction between amino acids and reducing sugars, as well as the degradation of amino acids and thiamines (Wang et al. 2021). Disulfide, dimethyl, and dimethyl trisulfide have sulfur‐ and onion‐like aroma characteristics (Ha et al. 2019). The content of these substances was the most abundant in yak meat obtained at a slaughter age of 2–4 years, likely due to the decrease in flavor‐producing amino acids with the increase in slaughter age (Zi et al. 2004). Choi et al. (2023) also reached a similar conclusion.

#### 3.3.2 Statistical Analysis of Volatile Components in Yak Meat From Different Slaughter Ages

**FIGURE 3:**
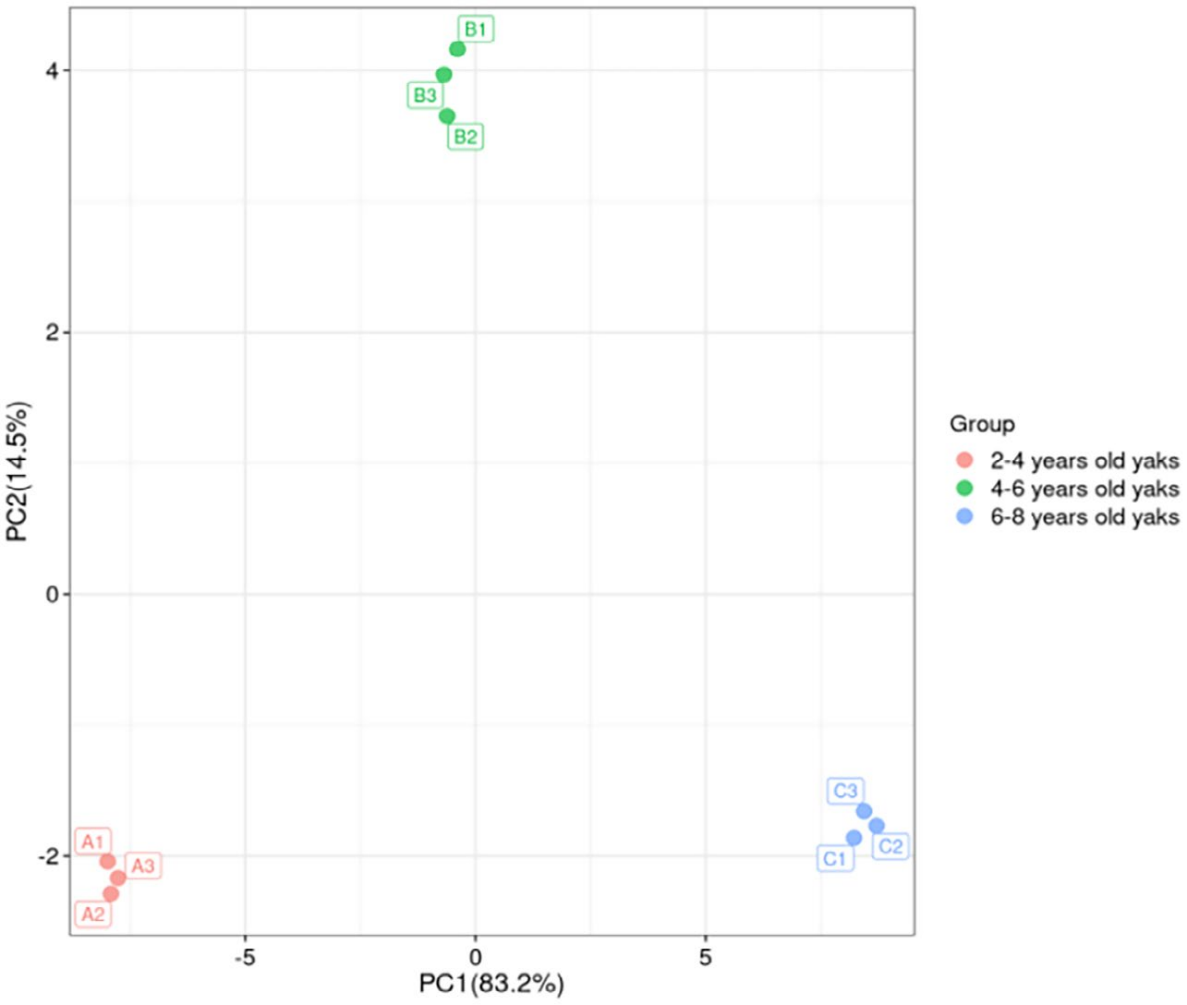
PCA diagram of volatile substances in *longissimus dorsi* muscle of Gannan yak at different slaughter ages.

**FIGURE 4:**
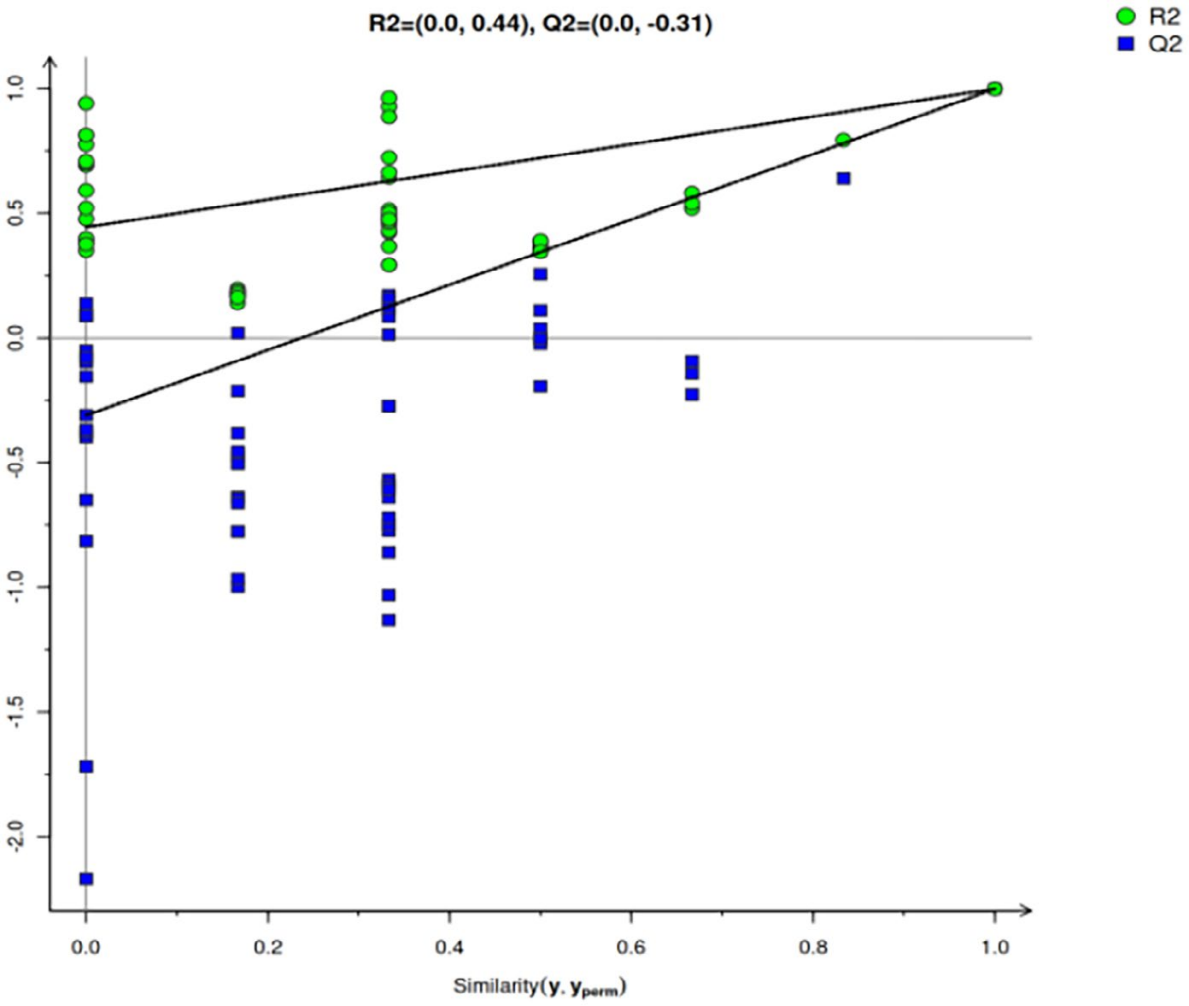
PLS‐DA replacement test results of volatile substances in *longissimus dorsi* muscle of Gannan yak at different slaughter ages.

Among the various multivariate methods available for analysis, PLS‐DA can maximize the difference between groups according to a predefined classification parameter (*Y* variable). Its discriminant effect is significant, and it can directly reflect the relationship between samples (Jandrić and Cannavan 2017). As shown in Figure 3, Gannan yak meat samples obtained at different slaughter ages showed obvious distribution patterns along the first principal component (PC1) in principal component analysis. Specifically, yak meat samples from the 2 to 4‐year slaughter group were mainly concentrated in the third quadrant, while those from the 4 to 6‐year slaughter group were mainly concentrated in the first quadrant. Additionally, yak meat samples from the 6 to 8‐year slaughter group were widely distributed in the fourth quadrant. Cluster analysis revealed that the intergroup distance between the samples was large, indicating that the content of volatile flavor substances was significantly altered with the increase in slaughter age (*p* < 0.05). Subsequently, the PLS‐DA model was cross‐validated. As shown in Figure 4, after 200 permutation tests, the explanatory power (R2X) of the model was 0.977, the predictive power (R2Y) was 0.999, and the predictive accuracy (Q2) was 0.999. The regression line of Q2 intersected the ordinate, and this point of intersection was below the origin, indicating that the model has good verification effect and there is no overfitting or underfitting. Therefore, the model appeared to be suitable for the identification and analysis of volatile components in yak meat obtained at different slaughter ages.

**FIGURE 5:**
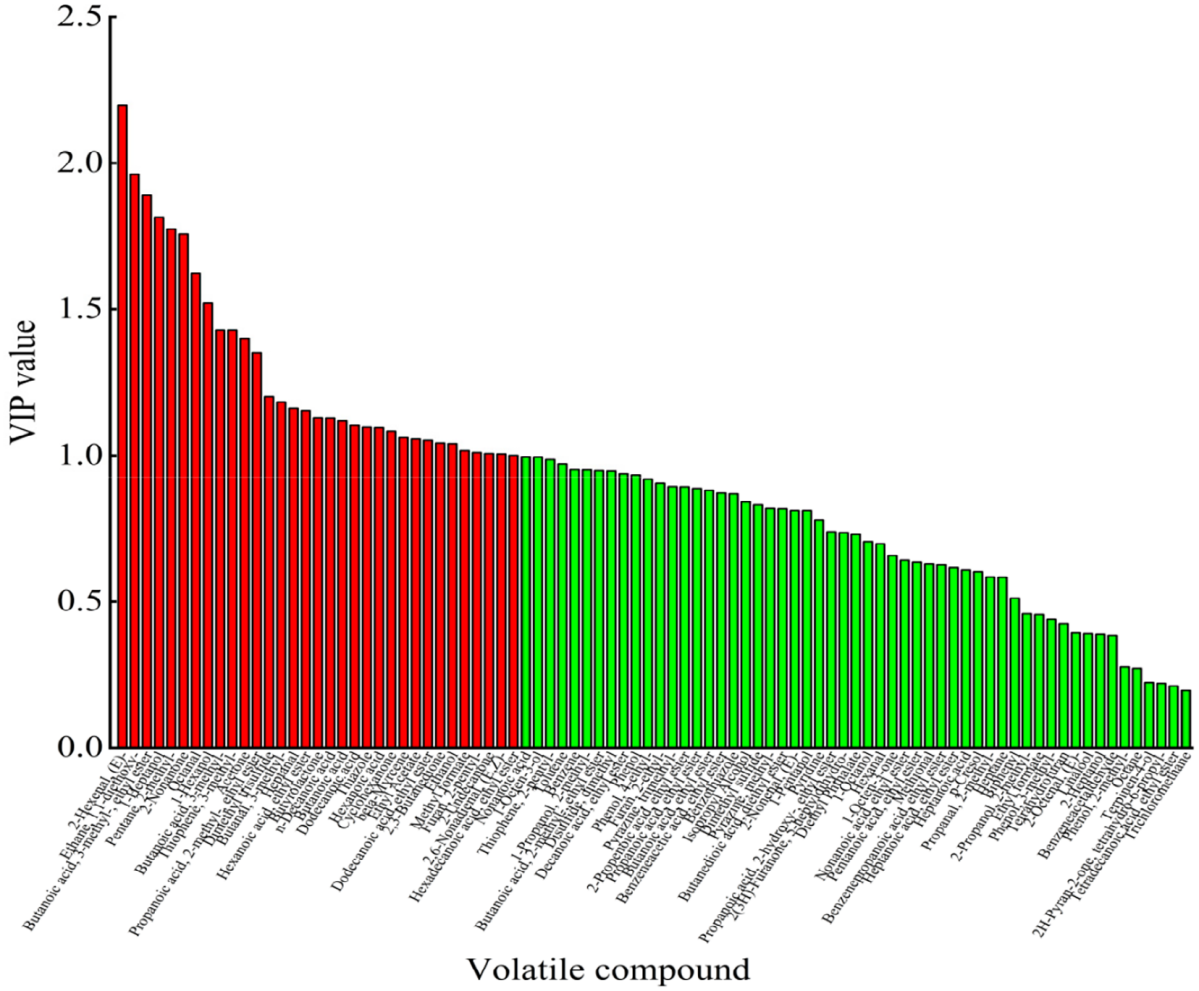
The VIP value of volatile substances in the longissimus dorsi muscle of Gannan Yak at different slaughter ages (red region representation: *p* < 0.05 and VIP > 1).

**FIGURE 6:**
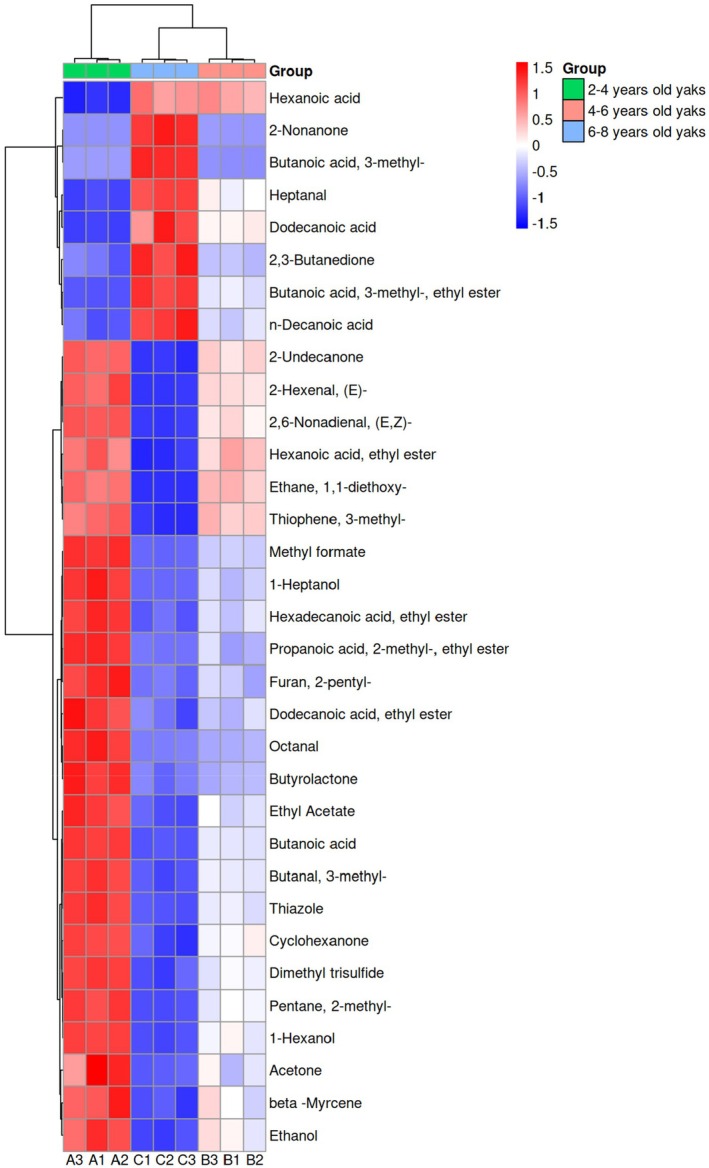
Cluster heat map of volatile differential substances in *longissimus dorsi* muscle of Gannan yak at different slaughter ages.

VIP is an important index for measuring the contribution of different compounds to model classification. When the VIP value exceeds 1, the variable may act as a differential parameter in the classification model, and the VIP value reflects the degree of difference across various groups of samples (Diler et al. 2022). To further study the contribution of volatile components to the flavor of yak meat, based on GC × GC‐ToF‐MS data, differential substances significance (*p* < 0.05 and VIP > 1) screened out. Figure 5 shows that a total of 33 compounds met these criteria, including eight esters, five aldehydes, five ketones, four organic acids, four organic heterocyclic compounds, three alcohols, two hydrocarbons, and one ether. In addition, the difference in volatile compounds across different slaughter ages was examined using a cluster heat map based on these criteria, with the depth of color representing the level of each volatile substance. As shown in Figure 6, the content of differential volatile compounds was significantly higher at a slaughter age of 2–4 years than at other slaughter ages. In particular, the relative contents of methyl formate, hexadecanoic acid, ethyl ester, and propanoic acid, 2‐methyl‐, ethyl ester were relatively high. These compounds can thus serve as potential markers to identify Gannan yak meat of different ages.

#### 3.3.3 Key Aroma Components in Gannan Yak Meat From Different Slaughter Ages

To conduct a more thorough evaluation of how various volatile substances impact the aroma of Gannan yak meat, ROAVs were employed as a metric. As shown in Table 7, a total of nine key odor‐active substances with ROAVs ≥ 1 were screened out from the volatile compounds present in Gannan yak meat at different slaughter ages. These included three aldehydes and ketones and one ester, heterocyclic, and sulfur‐containing compound. Notably, yak meat obtained at a slaughter age of 2–4 years had nine key odor‐active substances with ROAVs ≥ 1, namely, 2‐nonenal, (E)‐, 2‐octenal, (E)‐, heptanal, hexadecanoic acid, ethyl ester, 1‐octen‐3‐one, 2‐undecanone, 2,3‐butanedione, furan, 2‐pentyl‐, and disulfide, dimethyl. Meanwhile, yak meat obtained at a slaughter age of 4–6 years old contained seven key odor‐active substances with ROAVs ≥ 1, i.e., 2‐nonenal, (E)‐, 2‐octenal, heptanal, 1‐octen‐3‐one, 2‐undecanone, 2,3‐butanedione, and furan, 2‐pentyl‐. Finally, there were five key odor‐active substances with ROAVs ≥ 1 in yak meat obtained at a slaughter age of 6–8 years, that is, 2‐nonenal, (E)‐, 2‐octenal, heptanal, 2,3‐butanedione, and furan, 2‐pentyl‐. Among the aldehydes, 2‐nonenal, (E)‐(which had fat flavor) showed the highest ROAV, and its threshold was low. 2‐nonenal, (E)‐acted as a key aroma substance and was common to Gannan yak meat of different slaughter ages (Mariutti and Bragagnolo 2017). Among the ketones, 2,3‐butanedione (with pleasant and buttery odor that makes the overall flavor of the product fuller) was also identified as a key aroma substance common to different groups of yak meat. Furan, 2‐pentyl‐, which is a flavoring substance with meat and fruit flavor, also showed a high ROAV (1.36–10.04) (Zhang et al. 2020). Meanwhile, hexadecanoic acid, ethyl ester—the most representative short‐chain fatty acid esters with a strong fruity aroma (Watanabe et al. 2015)—and disulfide, dimethyl—with an onion, meat, and grease flavor (Ha et al. 2019) —emerged are key aroma substances in 2–4‐year‐old yak meat. They showed ROAVs greater than 1 only in this slaughter age group, simply playing a modifying role in meat from yaks aged 4–6 or 6–8 years. These findings demonstrated that yak meat obtained at a slaughter age of 2–4 years has more complex and distinct flavor characteristics.

**TABLE 7:** Key aroma components in *longissimus dorsi* muscle of Gannan yak at different slaughter ages.

Number	Volatile compounds	Thresholds^1^	Odor description^2^	A/ROAV	B/ROAV	C/ROAV	*p*
1	2‐Nonenal, (E)—	0.0002	Fatty, Cucumber	19.36 ± 2.89^a^	12.59 ± 0.23^b^	3.58 ± 0.25^c^	***
2	2‐Octenal, (E)—	0.003	Nuts, Green, Fatty	6.07 ± 0.85^a^	3.97 ± 0.14^b^	1.87 ± 0.11^c^	***
3	Heptanal	0.003	Citrus, Fatty, Rancid	11.29 ± 1.67^c^	18.33 ± 1.37^a^	14.07 ± 0.87^b^	**
4	Hexadecanoic acid, ethyl ester	2	Wax	1.19 ± 0.18^a^	0.51 ± 0.04^b^	0.17 ± 0.02^c^	***
5	1‐Octen‐3‐one	0.005	Mushroom‐Like	1.83 ± 0.17^a^	1.02 ± 0.05^b^	0.43 ± 0.06^c^	***
6	2‐Undecanone	0.004355	Orange, Fresh, Green	6.97 ± 0.99^a^	4.01 ± 0.24^b^	0.94 ± 0.08^c^	***
7	2,3‐Butanedione	0.002	Pleasant, Buttery	100.00 ± 0.00	100.00 ± 0.00	100.00 ± 0.00	ns
8	Furan, 2‐pentyl—	0.006	Green Beans, Vegetable	10.04 ± 1.84^a^	3.75 ± 0.29^b^	1.36 ± 0.15^c^	***
9	Disulfide, dimethyl	0.29	Garlic, Putrid, Asparagus	1.39 ± 0.24^a^	0.62 ± 0.05^b^	0.21 ± 0.03^c^	***

*Note:* Yaks aged 2–4 years (A), 4–6 years (B), and 6–8 years (C); ^a,b,c^Means within a row with different superscripts differ significantly (*p* ≤ 0.05); ns = *p* > 0.05; ⁎*p* ≤ 0.05; ⁎⁎*p* ≤ 0.01; ⁎⁎⁎*p* ≤ 0.001.

^1^ The threshold value of volatile flavor substances refers to the minimum concentration of aroma compounds that can be felt by human olfactory organs. The data are derived from Djoumbou Feunang et al. (2016).

^2^ The odor description comes from Garg et al. (2018).

## 4 Conclusions

In summary, meat from 2 to 4‐year‐old yaks showed the best quality in terms of sensory properties, texture characteristics, and flavor composition. Specifically, the tenderness, juiciness, and flavor scores of yak meat were significantly better when the slaughter age was 2–4 years than at other slaughter ages. In addition, the water loss rate, cooking loss, and drip loss were also the lowest in this group, while the WHC was the highest. The texture characteristics (such as hardness, chewiness, elasticity, etc.) of yak meat obtained at a slaughter age of 2–4 years were better aligned with consumer preferences. From a nutritional perspective, a slaughter age of 2–4 years also provided meat with the most balanced FAA composition, with a higher content of umami and sweet amino acids and lower content of bitter amino acids. In terms of volatile flavor substances, the content of key flavor substances (such as 2‐nonenal, (E)‐, 1‐octen‐3‐one, hexadecanoic acid, ethyl ester and disulfide, and dimethyl) was significantly higher in meat from yaks aged 2 to 4 years than in meat from other age groups, providing more complex and distinct primary flavor characteristics. Furthermore, the content of UFA and the PUFA/SFA ratio were higher in meat from yaks aged 2 to 4 years, and the nutritional value was superior. In summary, meat obtained from Gannan yaks aged 2 to 4 years showed significant advantages in terms of sensory quality, texture characteristics, nutritional components, and flavor composition and was more suitable for processing into high‐quality meat products. This study provides a scientific basis for selecting the slaughter age of yaks and optimizing yak meat quality and is of considerable significance for improving the market competitiveness and consumer favorability of yak meat. Although this study comprehensively evaluated the meat quality characteristics of yaks at different ages and determined that 2–4 years old was the best slaughter age, there were still some limitations in the study. First, the study is mainly based on yak samples in specific regions, which may have regional limitations. The optimal slaughter age of yaks in other regions may be different due to differences in feeding conditions. Second, meat quality characteristics are affected by many factors, such as feeding management, genetic background, etc. This study mainly focused on slaughter age, and other factors were not discussed in depth. Finally, the research mainly starts from the perspective of meat quality. In the future, it can provide a more comprehensive decision‐making basis for the yak industry in combination with multi‐dimensional factors such as economy and market.

## Author Contributions

**Xin Yu:** data curation (equal), investigation (equal), methodology (equal), writing – original draft (equal), writing – review and editing (equal). **Caiyun Li:** supervision (equal). **Ziyi Zhao:** formal analysis (equal), resources (equal). **Yubin Zhang:** supervision, conceptualization.

## Disclosure

Institutional Review Board Statement: The animal experiment was approved by the Ethics Committee of Gansu Agricultural University (License No.: GSAU‐Eth‐FSE‐2024‐006).

## Conflicts of Interest

The authors declare no conflicts of interest.

## Data Availability

The original contributions presented in the study are included in the article; further inquiries can be directed to the corresponding authors.
